# Non-Parametric Change-Point Method for Differential Gene Expression Detection

**DOI:** 10.1371/journal.pone.0020060

**Published:** 2011-05-31

**Authors:** Yao Wang, Chunguo Wu, Zhaohua Ji, Binghong Wang, Yanchun Liang

**Affiliations:** 1 Key Laboratory for Symbol Computation and Knowledge Engineering of National Education Ministry, College of Computer Science and Technology, Jilin University, Jilin, China; 2 Inner Mongolia Xing'an Vocational & Technical College, Wulanhaote, China; 3 Business School, University of Shanghai for Science and Technology, Shanghai, China; 4 Department of Modern Physics, University of Science and Technology of China, Hefei, China; Health Canada, Canada

## Abstract

**Background:**

We proposed a non-parametric method, named Non-Parametric Change Point Statistic (NPCPS for short), by using a single equation for detecting differential gene expression (DGE) in microarray data. NPCPS is based on the change point theory to provide effective DGE detecting ability.

**Methodology:**

NPCPS used the data distribution of the normal samples as input, and detects DGE in the cancer samples by locating the change point of gene expression profile. An estimate of the change point position generated by NPCPS enables the identification of the samples containing DGE. Monte Carlo simulation and ROC study were applied to examine the detecting accuracy of NPCPS, and the experiment on real microarray data of breast cancer was carried out to compare NPCPS with other methods.

**Conclusions:**

Simulation study indicated that NPCPS was more effective for detecting DGE in cancer subset compared with five parametric methods and one non-parametric method. When there were more than 8 cancer samples containing DGE, the type I error of NPCPS was below 0.01. Experiment results showed both good accuracy and reliability of NPCPS. Out of the 30 top genes ranked by using NPCPS, 16 genes were reported as relevant to cancer. Correlations between the detecting result of NPCPS and the compared methods were less than 0.05, while between the other methods the values were from 0.20 to 0.84. This indicates that NPCPS is working on different features and thus provides DGE identification from a distinct perspective comparing with the other mean or median based methods.

## Introduction

When normal gene expression is exposed to radiation, virus infection, etc., it would cause gene mutation or gene abnormal activation, which probably leads to cancer arising [1]. There are observable differences between cancer and normal tissues in their expression values on single-gene level, which enables recognition of cancer-related gene from a statistical perspective.

Based on microarray gene expression profiling [2], many methods were reported aiming to detect such difference in gene expression, or normally called differential gene expression (DGE) [3], [4]. Among these methods, T-statistics is a classical and widely-used DGE detecting methods, which works on the hypothesis that all the cancer samples are over-expressed compared with the normal samples [5]. Other work has also presented meaningful results, such as empirical Bayes approach [6] (Efron 2001), mixture model approach [7] (Pan, 2003), and SAM [8] (Storey 2003). However, considering the heterogeneity of gene activation, it is reasonable to assume that DGE could only take place in a subset of cancer samples. Many methods were proposed to solve DGE detection under this assumption, such as PPST (permutation percentile separability test) [9] (Lyons-Weiler, 2004), COPA (cancer outlier profile analysis) [10], [11], OS (outlier sum) [12] (Tibshirani, 2007), ORT (outlier robust t-statistics) [13] (Wu, 2007), and MOST (maximum ordered subset t-statistics) [14] (Lian, 2008).

Most of the aforementioned methods attempt to identify the abnormal data points based on the overall percentile of the gene expression profile. However, it is reasonable to assume that the DGE detection could be achieved by searching for the change point of the gene expression profile If we consider the single-gene expression profile as a data sequence, for non-DGE sequence, there is no significant change between the data distributions of normal and cancer samples; for DGE sequence, since the gene expression is over regulated in cancer group, the data distribution of cancer and normal samples become distinctly different, which would result in a significant change point in the sequence of gene expression profiles.

Change point problem [15] was widely studied in many fields, such as atmospheric and financial analysis. There are also applications of change-point theory to the microarray analysis, for example, a change point detection model for genomic sequences of continuous measurements [16], ARTIVA formalism for topology inference of regulatory network [17], a Bayesian model for DGE patterns of the DosR regulon of Mycobacterium tuberculosis in the timing of gene induction [18]. With respect to DGE analysis, there are BRIDGE (Bayesian robust inference for differential gene expression) for DGE detection in microarrays with small sample sizes [19], and DGE detecting method LRS (likelihood ratio test) [20] (Hu, 2008).

Since a few of the currently available change-point methods deal explicitly with estimation of the number and location of change points, and moreover these methods may be somewhat vulnerable to deviations of model assumptions usually employed [16], we propose a non-parametric statistical method for DGE detection, named as NPCPS (Non-Parametric Change Point Statistics). NPCPS is based on modified Kolmogorov statistic to detect the single-change point in a data sequence [21]. This method compares the data distribution of normal and cancer group to detect the existence of possible change-point in the cancer group, and to estimate the position of change-points. Besides, as a non-parametric inferential method, NPCPS does not make assumptions about the probability distributions of the variables being assessed, and accordingly, it is not necessary to normalize the microarray data before calculating the test statistic like other parametric methods usually do. As comparison, we tested several percentile-based methods and LRS. BRIDGE was not included as it is originally designed for two-sample problem and application to larger sample size is computationally heavy. NPCPS works comfortably with large-scale dataset, and both simulation and experiment results show that NPCPS is effective for DGE detection.

## Methods

Suppose 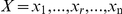 are independent random variables with cumulative distribution function 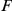, and *r* is the change point of 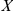. Then, for distribution function 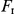 of 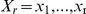 and 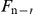 of 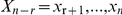, there exists a value 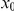 that satisfies 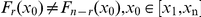. Otherwise, if *r* is not the change point, we have 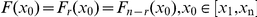. The change point is also noted as
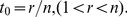
(1)


Gene expression profile of a single gene could also be considered as a sequence of independent variables as below:
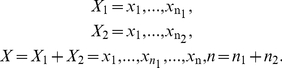
(2)Here, 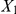 contains expression values of normal samples in known distribution function 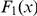, and 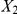 contains expression values of disease samples. Over or under expression values in 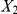 would result in a change point in 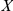.

To detect the change point, in the hypothesis test we used a modified Kolmogorov statistic (K-statistic), which evaluates the distance between two distribution functions:
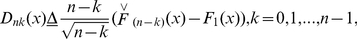
(3)

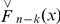
 is the empirical distribution function of 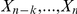, defined as:
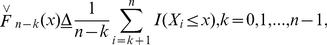
(4)where 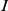 is an indicator function. 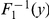 is the inverse function of 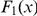 defined as
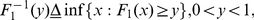
(5)where *y* is a variable increasing with a fixed step that is subject to user's selection (we selected 100 in the simulation study and experiment).

Therefore, the testing procedure is defined as
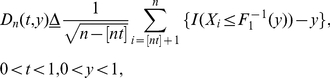
(6)where 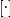 means round toward negative infinity.

Null hypothesis 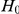 is true when 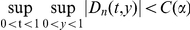, i.e. no change point detected; alternative hypothesis 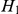 is true when 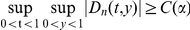, i.e. 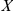 has a change point. 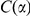 is the critical value and α is the significance level. Typical values of 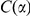 include *C*(0.05) = 1.358 and *C*(0.05) = 1.628.

To give an estimate of change point, we define
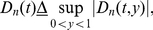
(7)and
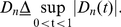
(8)Let 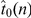 be the estimate of 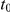, which is defined as:
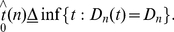
(9)Since the test statistic measures the difference between two distribution functions, larger 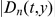 indicates more significant DGE, while the positive 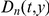 corresponds to under-expression and the negative 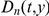 corresponds to over-expression, respectively.

## Results and Discussion

### Methods compared with NPCPS

Gene expression profile obtained from microarray data is often considered as a *g*×*n* matrix, which contains *g* rows of genes with their expression levels in *n* samples, in which normal group has *n*_1_ samples, and disease group has *n*_2_ samples. Let *x_ij_* be the expression intensity of the *i*th gene in the *j*th sample of the normal group, while *i* = 1, 2, …, *g*, *j* = 1, 2, …, *n*_1_; let *y_ij_* be the expression intensity of the *i*th gene in the *j*th sample of the disease group, while *i* = 1, 2, …, *g*, *j* = 1, 2, …, *n*_2_. The median of the *i*th gene is defined as
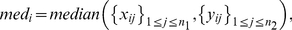
(10)Define *med_ix_*, the normal-group median of the *i*th gene, and *med_iy_* the cancer-group median as
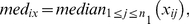
(11)

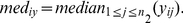
(12)


#### Parametric methods for DGE in cancer subset

There have been many parametric methods proposed based on the mean, median and median absolute deviation (MAD) of the gene expression profile, and following are some typical methods.

**COPA** [10], [11]: COPA first normalizes the expression data using the group mean and MAD to prevent impact to the data distribution hypothesis by outliers, then sorts the expression value and detects cancer genes though the rth percentile of the cancer group. If the MAD of the ith gene is approximated as
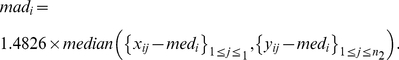
(13)The COPA statistic is defined as
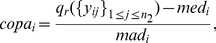
(14)where *q_r_*(*i*) is the *r*th percentile of the *i*th gene expression values, which is subject to the user's selection.

**OS** [12]: OS introduced heuristic rule as an additional function, and also applied the percentile knowledge to detect DGE. OS normalizes every gene to ensure the same data scale, which is convenient for gene comparison. In OS, gene expression values greater than *Q*_3_(*i*)+*IQR*(*i*) and smaller than *Q*_1_(*i*)−*IQR*(*i*) are statistically considered as DGE. The OS statistic is defined as below.
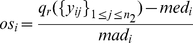
(15)The group with over expression is defined as
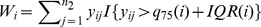
(16)Similarly, the group with under expression DGE is defined as
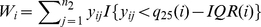
(17)


**ORT** [13]: Compared with OS, ORT is similar but uses the median of the normal group instead of the median of all data, and estimates the absolute error using the median of several groups instead of the square error as in COPA [11]. The purpose of these changes is to acquire more robust and consistent estimation. Accordingly, the estimate MAD in ORT is
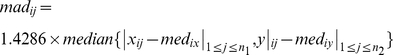
(18)The ORT statistic is
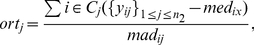
(19)where *C_i_* is the cancer group of the *i*th gene. For over expression,
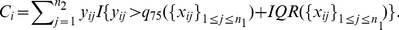
(20)Similarly, for under expression,
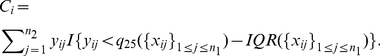
(21)


**MOST** [14]: Gene with expression value greater than *MOST_ik_* is considered as a differential gene expression. The testing statistic *MOST_ik_* is defined as:
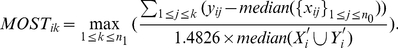
(22)When *k* is unknown, the data are normalized firstly by *μ_k_* and *σ^2^_k_*, and the *MOST_ik_* is defined as
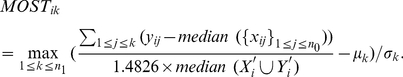
(23)


#### Non-parametric methods for DGE in cancer subset

**PPST** [9]: As a non-parametric method, PPST compares the expression levels of thousands of genes in two sample groups, i.e. the control group (A) and the case group (B). The detection focuses on genes in group A of which the expression levels are higher than a certain percentile of group B's expression values (A>B), which is a type I error in statistics, and vice versa (B>A). There are two marks for each gene, s1 and s2. S1 is the number of samples in group A that are higher than the 95% of group B added by the number of samples in group B that are lower than the 95% of group A. S2 is defined as the opposite to s1. Considering a given gene, if the expression value in group A is higher than the 95th percentile of group B, it is considered as over expression; if the expression value is lower than that in group B, it is considered as under expression. Define the PPST statistic of each gene with over expression as:
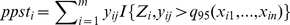
(24)PPST statistic of gene with under expression can be obtained similarly.

**LRS** [15]: in LRS, cancer outlier samples are viewed as coming from a distribution with higher mean expression intensity than all the normal and other cancer samples. The purpose of LRS is to test such unequal mean. For up-regulation, LRS first organizes all the samples so the non-cancer samples are arranged before the cancer samples, and the cancer samples are sorted by their expression intensities in the ascending order. *S_n_* is the summation of the expression intensities of all the samples and the LRS statistic is as follows,
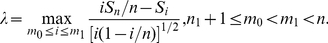
(25)


#### Traditional methods for DGE in entire cancer group

**T-statistic** [5]: this traditional method assumes that the cancer sample group is generally over or under-expressed compared with the normal samples. The t-statistic is defined as:
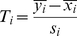
(26)where 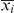 is the sample mean of normal group expression values and 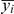 is the sample mean of cancer group expression values, *S_i_* is the estimate of combined standard deviation. Differentially expressed genes are recognized when the testing statistic exceeds a certain threshold.

### Monte Carlo simulation

**Figure 1:**
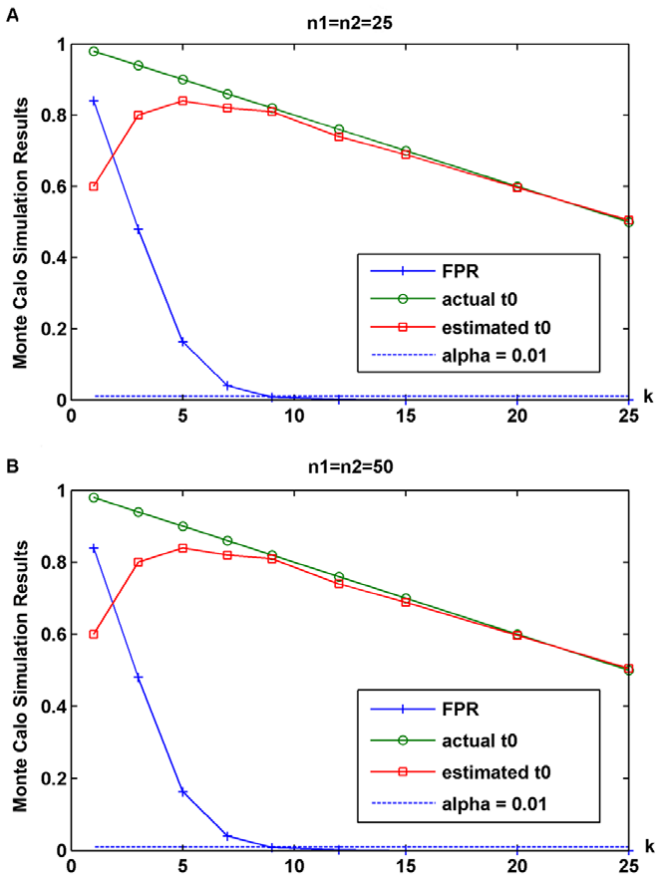
FPR and estimate of change-point position. (A) Monte Carlo simulation results of dataset with size n_1_ = n_2_ = 25 and significance level α = 0.01. (B) Monte Carlo simulation results of dataset with size n_1_ = n_2_ = 50 and significance level α = 0.01. The x-axis is *k*, the number of samples in simulated dataset that contained DGE. The trend of curves in (A) and (B) was similar. Both FPR and estimate of change-point enhanced with the increasing *k*. When *k*>9, the difference between the true change-point and the estimated change-point was very small, and the FPR of NPCPS became lower than the significance level α, which indicated that the hypothesis test of NPCPS passed the Monte Carlo simulation.

Monte Carlo simulation can be used to evaluate the performance of a hypothesis test in terms of the ratio of Type I error, i.e. false positive rate (FPR). For each Monte Carlo simulation, NPCPS was applied to an artificial 7000-gene dataset (normal random numbers with mean = 0, standard deviation sd = 1) composed of n_1_ normal samples and n_2_ cancer samples, of which *k* (0<*k*<*n*_2_) cancer samples contained DGE simulated by adding a constant μ to the original normal random numbers. Multiple simulations were carried out according to different values of sample size *n*, DGE sample size *k*, and significance level α. The FPR (Table 1) and average estimate of change point (Table 2) were computed and the results of simulation with α = 0.01 were illustrated in Fig. 1. For data set *n*_1_ = *n*_2_ = 25 (Fig. 1A), the FPR was larger when *k* was smaller; FPR decreased with *k* increasing; when *k* was equal to or larger than 9, the detecting accuracy of NPCPS was sufficient to satisfy the significance level. For data set *n*_1_ = *n*_2_ = 50 (Fig. 1B), *k* should be not less than 9 to satisfy the significance level. The estimate of change point enhanced greatly when *k* increased; the estimated position became very close to the actual position at the same time as the FPR dropped below the significance level. This indicates that NPCPS is highly sensitive to left boundary and less sensitive to the right boundary, when the *F*_2_ information is not sufficient [21].

**Table 1:** FPR of NPCPS in Monte Carlo simulation.

n_1_ = n_2_ = 25, α = 0.05, C(α) = 1.358									
*k*	1	3	5	7	9	12	15	20	25
**FPR**	0.5953	0.1524	0.0333	0.0054	0.0007	0.0001	0.0	0.0	0.0

n_1_ = n_2_ = 25, α = 0.01, C(α) = 1.628									
*k*	1	3	5	7	9	12	15	20	25
**FPR**	0.84	0.48	0.163	0.04	0.008	0.002	0.0	0.0	0.0

n_1_ = n_2_ = 50, α = 0.05, C(α) = 1.358									
*k*	1	4	7	9	12	17	22	30	50
**FPR**	0.5263	0.0541	0.0040	0.0006	0.0	0.0	0.0	0.0	0.0

n_1_ = n_2_ = 50, α = 0.01, C(α) = 1.628									
*k*	1	4	7	9	12	17	22	30	50
**FPR**	0.802	0.268	0.042	0.009	0.0	0.0	0.0	0.0	0.0

**Table 2:** Actual and average estimate of change point using NPCPS in Monte Carlo simulation.

n_1_ = n_2_ = 25, α = 0.01, C(α) = 1.628									
*k*	1	3	5	7	9	12	15	20	25
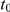	0.98	0.94	0.90	0.86	0.82	0.76	0.70	0.60	0.50
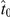	0.6	0.80	0.84	0.82	0.81	0.74	0.689	0.597	0.505

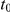
: Actual change point. 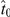: Average estimate of change point.

### ROC analysis on simulated data

**Figure 2:**
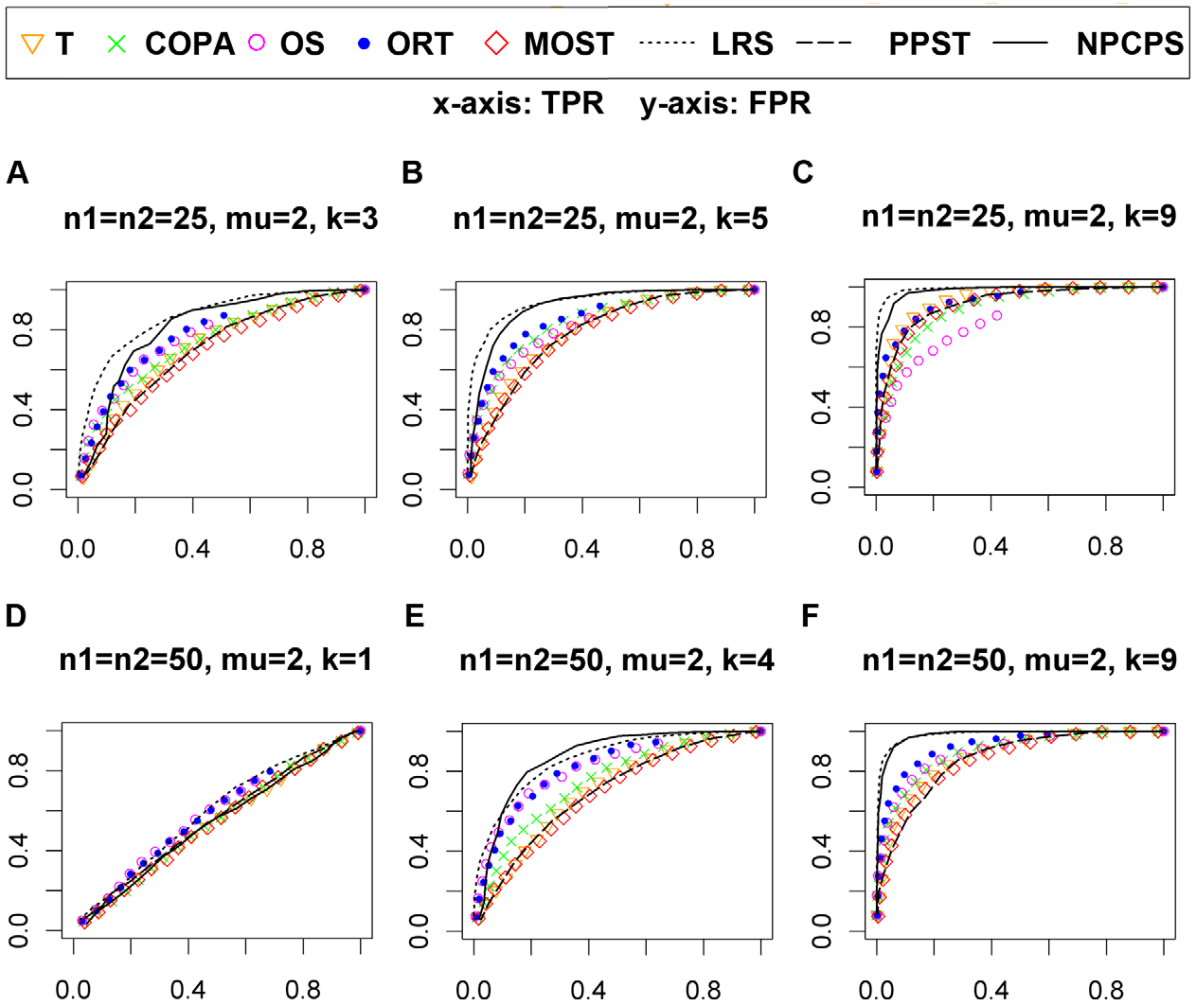
Selected ROC curves of normal dataset with μ = 2. (A) n_1_ = n_2_ = 25, *k* = 3. (B) n_1_ = n_2_ = 25, *k* = 5. (C) n_1_ = n_2_ = 25, *k* = 9. (D) n_1_ = n_2_ = 50, *k* = 1. (E) n_1_ = n_2_ = 50, *k* = 4. (F) n_1_ = n_2_ = 50, *k* = 9. The x-axis is FPR, and the y-axis is TPR. The significance level α = 0.01 for NPCPS. Larger area under ROC curves indicates better sensitivity and specificity. An ROC curve along the diagonal line indicates random-guess.

**Figure 3:**
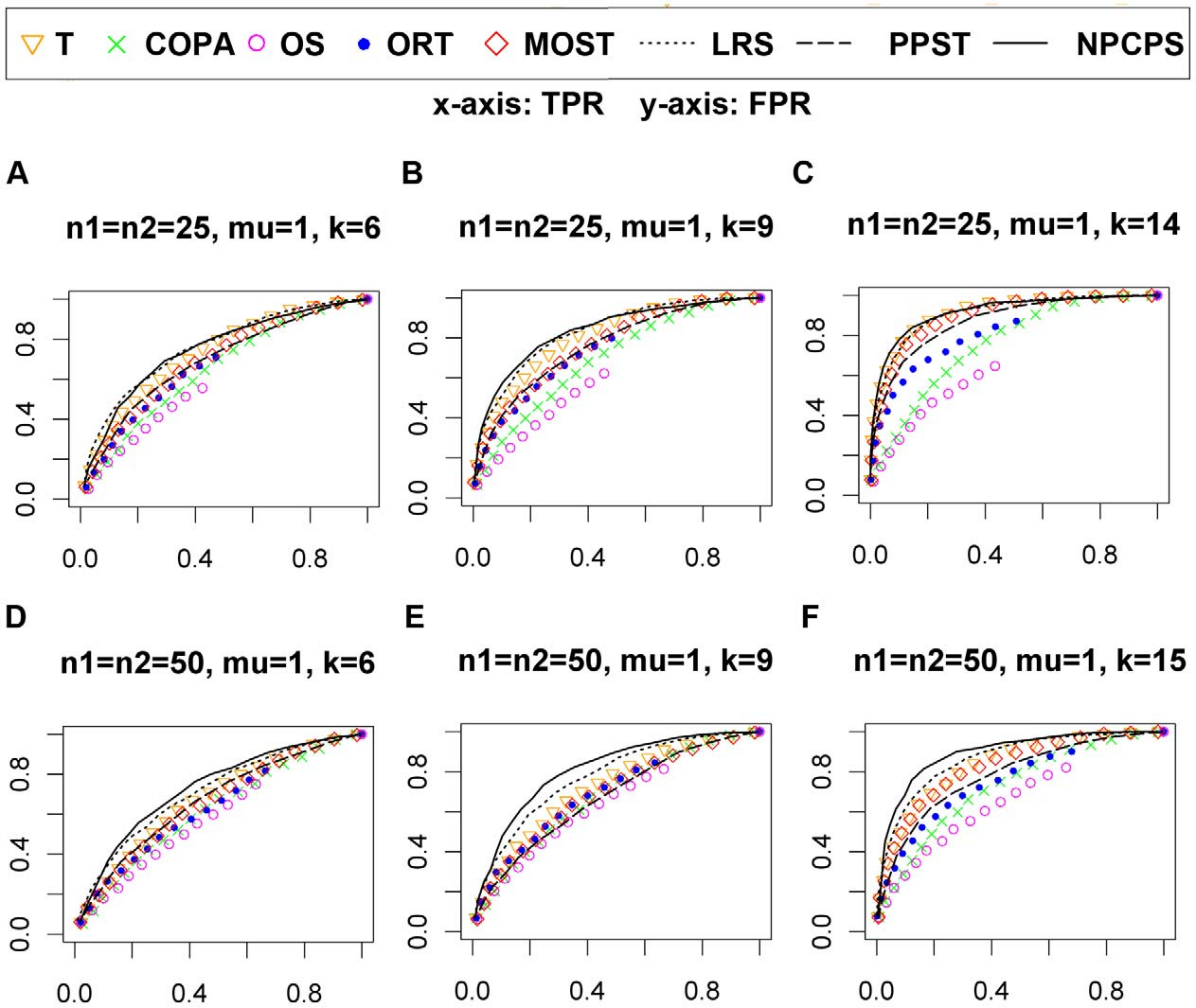
Selected ROC curves of normal dataset with μ = 1. (A) n_1_ = n_2_ = 25, *k* = 6. (B) n_1_ = n_2_ = 25, *k* = 9. (C) n_1_ = n_2_ = 25, *k* = 14. (D) n_1_ = n_2_ = 50, *k* = 6. (E) n_1_ = n_2_ = 50, *k* = 9. (F) n_1_ = n_2_ = 50, *k* = 15. The x-axis is FPR, and the y-axis is TPR. The significance level α = 0.01 for NPCPS. Larger area under ROC curves indicates better sensitivity and specificity. An ROC curve along the diagonal line indicates random-guess.

First, we test NPCPS (α = 0.01) and seven other methods, namely COPA, ORT, OS, MOST, T, LRS, and PPST, on normally distributed datasets (mean = 0, sd = 1) with different μ, *n* and *k*. When *k* was getting greater, all methods produced better ROC (Fig. 2 and Fig. 3). For μ = 2, when *n* = 50 (Fig. 2A–2C), NPCPS was slightly weaker than LRS, and better than the other methods; when *n* = 100 (Fig. 2D–2E), NPCPS was very similar to LRS, and better than the other methods. For μ = 1, NPCPS gave the best performance for both *n* = 50 and *n* = 100 datasets and different values of *k* (Fig. 3A–3F). This indicated that NPCPS had better sensitivity for less significant DGE compared with the other seven methods. Among the non-parametric method, PPST was not significantly better than the parametric methods, while LRS and NPCPS were continuously better than the other methods. This indicated that methods based on change-point were more effective and robust than methods based on percentile and MAD.

**Figure 4:**
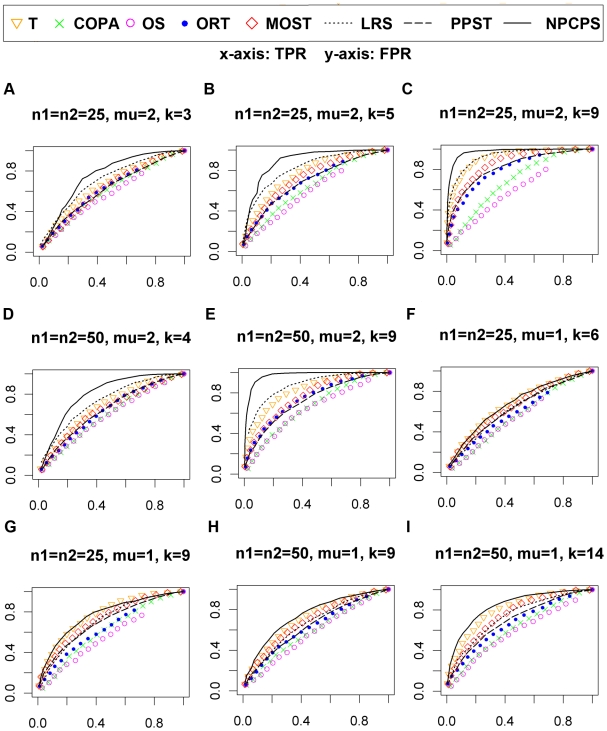
Selected ROC curves of skew-normal dataset. (A) n_1_ = n_2_ = 25, mu = 2, *k* = 3. (B) n_1_ = n_2_ = 25, mu = 2, *k* = 5. (C) n_1_ = n_2_ = 25, mu = 2, *k* = 9. (D) n_1_ = n_2_ = 50, mu = 2, *k* = 4. (E) n_1_ = n_2_ = 50, mu = 2, *k* = 9. (F) n_1_ = n_2_ = 25, mu = 1, *k* = 6. (G) n_1_ = n_2_ = 25, mu = 1, *k* = 9. (H) n_1_ = n_2_ = 50, mu = 1, *k* = 9. (I) n_1_ = n_2_ = 50, mu = 1, *k* = 14.The x-axis is FPR, and the y-axis is TPR. The significance level α = 0.01 for NPCPS. Larger area under ROC curves indicates better sensitivity and specificity. An ROC curve along the diagonal line indicates random-guess.

Second, we tested NPCPS (α = 0.01) and other seven methods on datasets generated from skew-normal (SN) distribution (Fig. 4). For different *n* and *k*, NPCPS had significantly larger area under the ROC curves compared with the other methods. By comparing Fig. 2 and Fig. 4 we can see that NPCPS was both effective for normal and skew-normal data distribution, and when *k* = 9 gave similarly good ROC (Fig. 2C compared with Fig. 4C, Fig. 2F compared with Fig. 4E). The other seven methods, including non-parametric methods, LRS and PPST, had inferior results when working with skew-normal data.

The AUC of ROC is summarized in Table 3 and 4.

**Table 3:** AUC of ROC curves of the simulation on data in normal distribution.

Data Parameter			AUC							
n	mu	k	NPCPS	LRS	COPA	OS	ORT	PPST	T	MOST
50	2	3	0.79	0.82	0.72	0.75	0.76	0.69	0.68	0.67
50	2	5	0.90	0.92	0.82	0.80	0.85	0.79	0.79	0.76
50	2	9	0.96	0.97	0.87	0.81	0.92	0.91	0.91	0.89
100	2	1	0.52	0.57	0.53	0.28	0.58	0.54	0.53	0.52
100	2	4	0.85	0.84	0.72	0.81	0.82	0.69	0.69	0.67
100	2	9	0.96	0.96	0.88	0.90	0.92	0.85	0.86	0.84
50	1	6	0.73	0.74	0.63	0.58	0.65	0.68	0.72	0.67
50	1	9	0.82	0.81	0.67	0.61	0.72	0.76	0.79	0.74
50	1	14	0.90	0.89	0.73	0.65	0.80	0.87	0.90	0.87
100	1	6	0.71	0.70	0.60	0.59	0.59	0.66	0.65	0.63
100	1	9	0.79	0.76	0.66	0.64	0.69	0.66	0.70	0.67
100	1	15	0.88	0.85	0.70	0.66	0.75	0.78	0.82	0.80

**Table 4:** AUC of ROC curves of the simulation on data in skew-normal distribution.

Data Parameter			AUC							
n	mu	k	NPCPS	LRS	COPA	OS	ORT	PPST	T	MOST
50	2	3	0.73	0.69	0.58	0.57	0.65	0.62	0.64	0.61
50	2	5	0.87	0.80	0.61	0.59	0.69	0.69	0.75	0.71
50	2	9	0.95	0.91	0.66	0.59	0.80	0.82	0.90	0.83
100	2	4	0.78	0.71	0.58	0.58	0.63	0.63	0.66	0.63
100	2	9	0.95	0.85	0.64	0.64	0.74	0.72	0.80	0.75
50	1	6	0.66	0.60	0.54	0.55	0.59	0.63	0.66	0.63
50	1	9	0.75	0.70	0.60	0.56	0.63	0.70	0.76	0.71
100	1	9	0.72	0.64	0.57	0.57	0.62	0.63	0.70	0.67
100	1	14	0.82	0.71	0.60	0.58	0.66	0.69	0.78	0.73

### DGE detection in breast-cancer microarray data

The microarray data used in the experiment are provided by West [22]. In their experiment, primary breast tumors (between 1.5 and 5 cm in maximal dimension) from the Duke Breast Cancer SPORE frozen tissue bank were selected and diagnosed as invasive ductal carcinoma. In each case, a diagnostic axillary lymph node dissection was performed. The final dataset includes 49 samples, 25 samples of which have negative lymph nodes and 24 samples with positive lymph nodes, used here as normal sample and cancer sample, respectively. Gene expression profile of 7129 genes was obtained through annotation package hu6800 [23]. The original gene expression values ranged from 34 to 43053 and were initialized to the range from 3.5 to 10.7. Seven detection method (t-statistic, COPA, OS, ORT, MOST, PPST and LRS) were applied to the initialized gene expression profile, while NPCPS was applied to the original data. The calculated test statistics of these 7129 genes by these methods were sorted in descending order.

**Figure 5:**
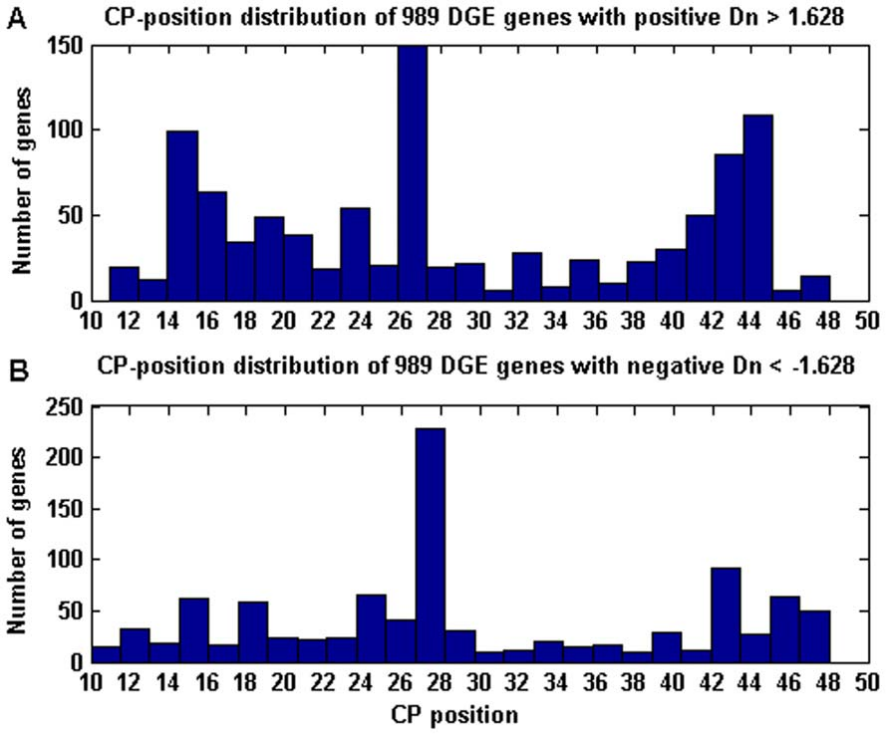
Change-point distribution of DGE genes when C(0.05) = 1.628. (A) CP-position distribution of 989 genes with positive *D* ***_n_***>1.628. (B) CP-position distribution of 989 genes with negative *D* ***_n_***<−1.628.

**Figure 6:**
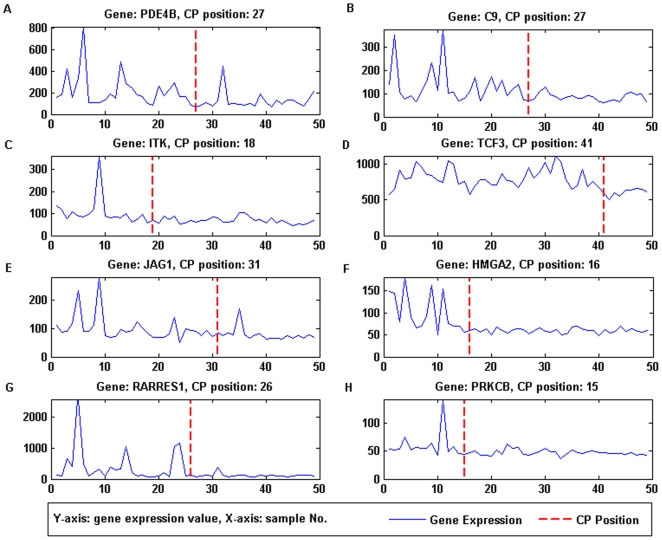
Expression value and Change-point of top-ranked DGE genes with positive *D_n_*. (A) PDE4B, change-point at sample 27. (B) C9, change-point at sample 27. (C) ITK, change-point at sample 27. (D) TCF3, change-point at sample 41. (E) JAG1, change-point at sample 31. (F) HMGA2, change-point at sample 16. (G) RARRES1, change-point at sample 26. (H) PRKCB, change-point at sample 15. CP position correctly locates the change in the trend of the gene expression value.

**Figure 7:**
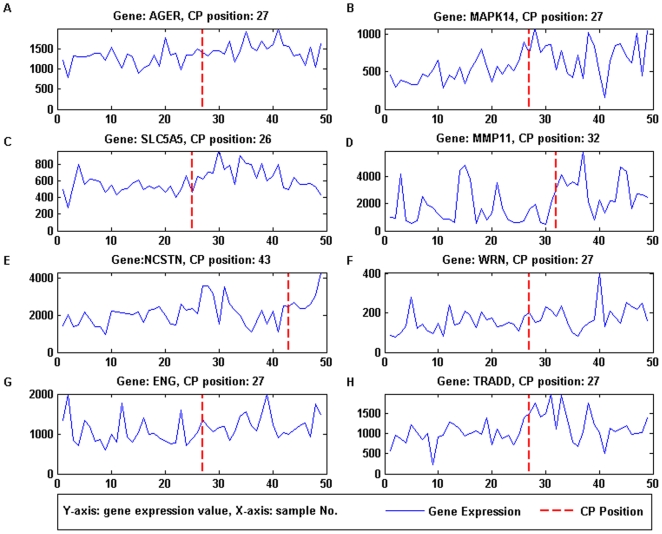
Expression value and Change-point of top-ranked DGE genes with negative *D_n_*. (A) AGER, change-point at sample 27. (B) MAPK14, change-point at sample 27. (C) SLC5A5, change-point at sample 26. (D) MMP11, change-point at sample 32. (E) NCSTN, change-point at sample 43. (F) WRN, change-point at sample 27. (G) ENG, change-point at sample 27. (H) TRADD, change-point at sample 27. CP position correctly locates the change in the trend of the gene expression value.

For NPCPS, *C*(0.05) = 1.628 was selected, which yields a detecting result of 1978 DGE genes. Fig. 5 shows the distribution of the estimated position of change-points in the expression value of these genes. We selected the first 30 genes ranked by NPCPS, and searched PubMed and other databases to confirm that whether these genes were relevant to breast cancer or other known cancers. Out of the first 30 genes identified by NPCPS, 17 have been reported as relevant to breast cancer or other cancers (as shown in Table 5 and Table 6 separately according to *D_n_* value). The gene expression values and the change-point (CP) positions of the cancer-relevant genes are illustrated in Fig. 6 and Fig. 7. From Fig. 6 and 7, it could be seen that the estimated change-point positions could successfully locate the change in the trend of the gene expression value.

**Table 5:** Results and description of top-ranked genes with positive test statistic *D_n_*.

NPCPS Ranking	Dn	CP Position	Gene Name	Description
1	2.91	27	PDE4B	The phosphodiesterase PDE4B limits cAMP-associated PI3K/AKT-dependent apoptosis in diffuse large B-cell lymphoma.
2	2.80	34	N/A	
3	2.79	32	N/A	
4	2.74	27	SCARB2	
5	2.74	27	C9	Upregulation of plasma C9 protein in gastric cancer patients.
6	2.73	22	RAB2A	
7	2.73	27	MEF2A	
8	2.69	37	N/A	
9	2.66	19	ITK	ITK-SYK causes a T-cell lymphoproliferative disease in mice, supporting its role in T-cell lymphoma development in humans.
10	2.66	41	TCF3	Misregulation plays a role in disease such as cancer, where overactive Wnt signaling drives LEF/TCFs to transform cells
11	2.64	31	JAG1	Heterogeneity of Jagged1 expression in human and mouse intestinal tumors: implications for targeting Notch signaling
12	2.64	16	HMGA2	LEF/TCFs to transform cells
13	2.62	28	NEFL	
14	2.60	16	IL2	The development of breast tumor is associated with an increased expression of IL-2 and this expression also seems to be associated with the malignancy of the tumor.
15	2.60	26	RARRES1	RARRES1 expression is significantly related to tumor differentiation and staging in colorectal adenocarcinoma
16	2.60	15	PRKCB	Target for inhibiting gastric cancer cell invasion
17	2.58	14	PRPS2	
18	2.56	19	IFNA10	

**Table 6:** Results and description of top-ranked genes with negative test statistic *D_n_*.

NPCPS Ranking	Dn	CP Position	Gene Name	Description
5336	−3.08	27	AGER	Serum sRAGE levels were influenced by genetic polymorphisms (−429 T/C, Gly82Ser and 2184 A/G) of the RAGE gene in breast cancer
1929	−2.91	27	GP1BB	
5918	−2.87	27		
2064	−2.87	27	MAPK14	The expression of p-p38 and uPA was negatively correlated to prognosis of breast cancer.
4931	−2.73	26	SLC5A5	The findings of this study indicated that NIS expression is prevalent in breast cancer brain metastases and could have a therapeutic role via the delivery of radioactive iodide and selective ablation of tumor cells
4634	−2.72	27	BMP1	
2124	−2.71	27		
5753	−2.69	27	MYOG	
5907	−2.62	32	MMP11	MMP-11 and CK-20 are probable prognostic markers whose expression reflects the stages of tumor differentiation and LNM of breast cancer
853	−2.60	43	NCSTN	NCSTN coded protein is a subunit of γ-Secretase compound, which is related to Notch signaling, a pathway found dysregulated in many cancers.
4869	−2.54	30	SLC4A2	
5060	−2.53	25	NAT6	
4128	−2.53	27	ALDH4A1	
4516	−2.52	21	SNAPC1	
2257	−2.51	27	WRN	The variant genotype of WRN Leu1074Phe was associated with a 1.36-fold significantly increased risk of breast cancer in Chinese women. This variant is also significantly associated with age at menarche
6187	−2.51	27	ENG	Elevated pretreatment plasma endoglin levels predicted for decreased clinical benefit and a shorter overall survival in metastatic breast cancer patients treated with 2nd-line hormone therapy.
2183	−2.51	27	TRADD	TRADD is involved in the p75(NTR)-mediated antiapoptotic activity of nerve growth factor in breast cancer cells
4444	−2.51	27	UNC119	

Moreover, by comparison among the ranking results of all eight methods based on the test statistic, it was noticed that genes top-ranked by NPCPS were ranked considerably lower by other methods, most of which were mean and median based parametric methods. When inspecting all the 7129 genes, the overall trend in ranking difference between NPCPS and other methods became more obvious. Table 7 shows the pair-wise linear correlation of gene ranking among the six methods. For NPCPS, the positive correlation is below 0.007 with OS, and the negative correlation below 0.047 with other methods. This indicated that NPCPS had much less correlation with the other five methods, among which the correlations were all positive and valued around 0.5 with each other. This will be further discussed in the following section. If NPCPS is combined with other methods, it would help to identify genes which are considered as less DGE significant by the other seven methods.

**Table 7:** Ranking relevance between each DGE detecting methods.

	NPCPS	LRS	COPA	OS	ORT	PPST	T	MOST
**NPCPS**	1	0.0354	−0.0346	0.0069	−0.0311	−0.0357	−0.0398	−0.0470
**LRS**	0.0354	1	0.5007	0.5274	0.3206	0.4182	0.4964	0.4474
**COPA**	−0.0346	0.5007	1	0.5339	0.5175	0.5825	0.5140	0.5752
**OS**	0.0069	0.5274	0.5339	1	0.4443	0.5407	0.5868	0.4539
**ORT**	−0.0311	0.3206	0.5175	0.4443	1	0.7921	0.2032	0.3069
**PPST**	−0.0357	0.4182	0.5825	0.5407	0.7921	1	0.3645	0.4378
**T**	−0.0398	0.4964	0.5140	0.5868	0.2032	0.3645	1	0.8394
**MOST**	−0.0470	0.4474	0.5752	0.4539	0.3069	0.4378	0.8394	1

### Discussions on the biological significance of NPCPS

#### Non-parametric statistics

As a non-parametric statistics, NPCPS does not rely on assumptions that the data are drawn from a given probability distribution. It is applicable to input data derived from various types distributions and doesn't require data pre-processing. As such it is the opposite of parametric statistics, which would have inferior performance when the input data are not in the assumed distribution, as in the ROC simulation on normal and skew-normal datasets (Fig. 4).

#### No restriction on both over expression and under expression

**Figure 8:**
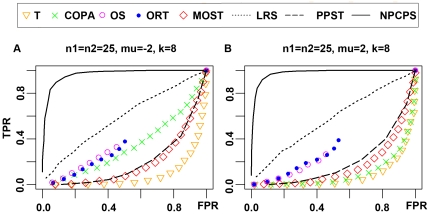
ROC curves of NPCPS and other methods when inappropriate formula applied. (A) Over expression formula applied to simulated under expressed gene, *n*_1_ = *n*_2_ = 25, μ = −2, *k* = 8. (B) Under expression formula applied to simulated over expressed gene, *n*_1_ = *n*_2_ = 25, μ = 2, *k* = 8. The x-axis is FPR, and the y-axis is TPR. ▽ is T, × is COPA, ○ is OS, • is ORT, ◊ is MOST, dotted line is LRS, dashed line is PPST, and solid line is NPCPS. The significance level α = 0.01 for NPCPS. NPCPS maintained the same level of sensitivity when applied to both types of simulated over-expressions. The other methods were not able to give results as good as when appropriate functions were applied as in Fig. 2 and Fig. 3.

The gene expression profile generated from microarray data usually contains samples of thousands of genes. Genes in the cancer samples might be over or under expressed. Majority of the DGE detecting methods have different formulas for under expressed and over expressed genes, respectively. For example, OS and ORT use different percentile values for over-expression and under-expression, respectively, and apply both formulas to the same microarray data. If over expression formula is applied to under expressed data, the DGE can not be correctly recognized. However, the detected results might contains false alarms, since both over-expression and under-expression formulas are applied to the same gene, and might be detected as DGE significant for twice. Unlike the other methods, NPCPS works for both types of DGE by using the same calculating formula, which would reduce the FDR, and do not require further analysis and computation aiming to clean the false alarms. When over expression formula was applied to under-expressed gene data (Fig. 8A), and vice versa (Fig. 8B), NPCPS presented stable performance in both situations, while other compared methods gave inferior ROC curves. According to the characteristic of ROC, T and MOST could have good ROC if the prediction result was inversed. The ROC curves of LRS were in the zone of random guess, which was close to the line-of-no-discrimination. Using LRS for under-expresson, user could turn under-expression into over-expression by inversing the dataset. This indicated that when over-expression formula of LRS was applied to under-expression, the random detecting result would be given.

### Estimated change point position: 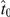


The biological meaning of 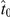 lies in that once the position of change point is estimated or located, we can identify which sample contains DGE. Then, rather than identifying DGE existence in *n* = *n*_1_+*n*_2_ samples on the single gene level, we can learn that, for one sample containing thousands of genes, how many genes were over expressed or under expressed. This statistical information can be used to analyze features of each sample, and the results of which could be applied to the estimation of the differentiation degree of cancer in different development stages.

### Distance between two distribution function: *D_n_*

**Figure 9:**
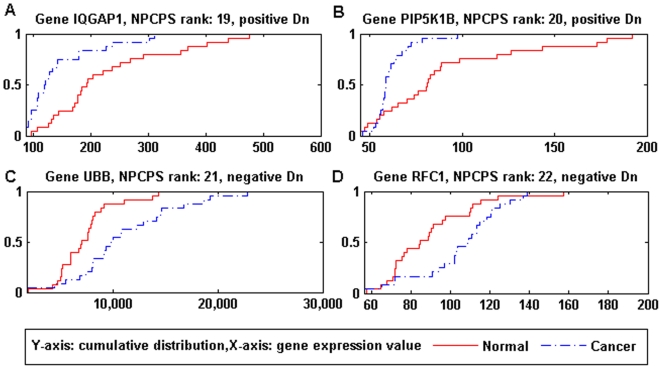
Data distributions of genes top-ranked by NPCPS. (A) I1GAP1: rank 19, positive *D_n_*. (B) PIP5K1B: rank 20, positive *D_n_*. (C) UBB: rank 21, negative *D_n_*. (D) RFC1: rank 22, negative *D_n_*. Top-ranked genes by NPCPS had significant difference between the data distributions of cancer and normal groups. By comparing the empirical distribution of cancer and normal samples, (A) and (B) had distributions of cancer group that were significantly left to the distribution of normal group, which demonstrated under-expression; (C) and (D) had distributions of cancer group that were significantly right to the distribution of normal group, which demonstrated over expression. The distribution curves were consistent with the biological significance of *D_n_* value.

**Figure 10:**
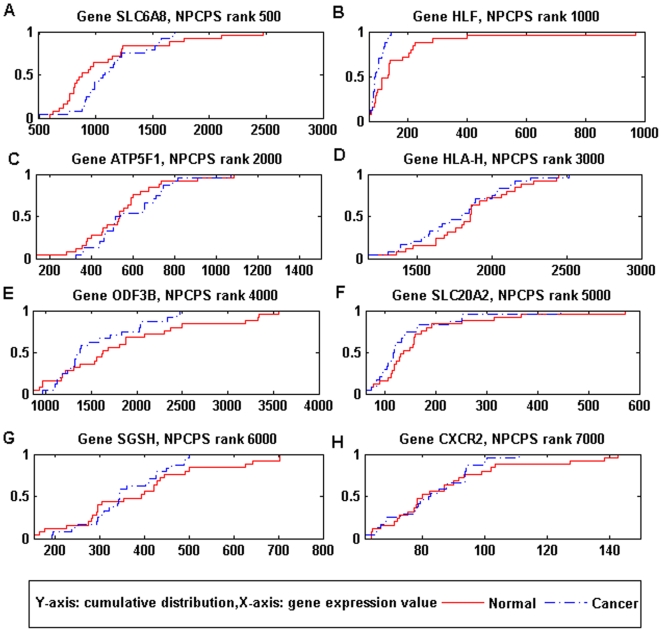
Data distribution of genes bottom-ranked by NPCPS. (A) SLC6A8: rank 500. (B) HLF: rank 1000. (C) ATP5F1: rank 2000. (D) HLA-H: rank 3000. (E) ODF3B: rank 4000. (F) SLC20A2: rank 5000. (G) SGSH: rank 6000. (H) CXCR2: rank 7000. From the empirical data distribution, the differences between cancer and normal groups in (A)–(D) were very small, which corresponded with the *D_n_* value.

NPCPS results showed that, among the 7219 genes, 3608 had negative *D_n_*, while the rest 3521 had positive *D_n_*. NPCPS use *D_n_* to evaluate the change in distribution between normal and cancer samples, and directly measure the DGE type as either over expressed or under expressed. This feature is valid based on the expression value in Fig. 6 and 7, where Fig. 6 (positive *D_n_*) shows typical under expression and Fig. 7 (negative *D_n_*) shows typical over expression. Fig. 9 and Fig. 10 can illustrate the relationship between *D_n_* and DGE in a more intuitive manner where cumulative data distributions of several typically ranked genes are given.

Genes in Fig. 9 were ranked on the top by NPCPS, where Fig. 9A and 9B are corresponding to positive *D_n_* (under-expression), 9C and 9D are corresponding to negative *D_n_* (over-expression), respectively. By comparing the empirical distribution of cancer and normal samples, in Fig. 9A and 9B, cancer group was significantly left to the normal group, which demonstrated under expression; in Fig. 9C and 9D, the cancer group was significantly right to the normal group, which demonstrated over-expression. The distribution graph was consistent with the *D_n_* value.

Genes in Fig. 10 were ranked lower by NPCPS. We can find that the cumulative distance between the data distributions of normal and cancer group is generally smaller compared with those genes top-ranked by NPCPS. From the empirical data distribution, difference between cancer and normal groups were very small.

**Figure 11:**
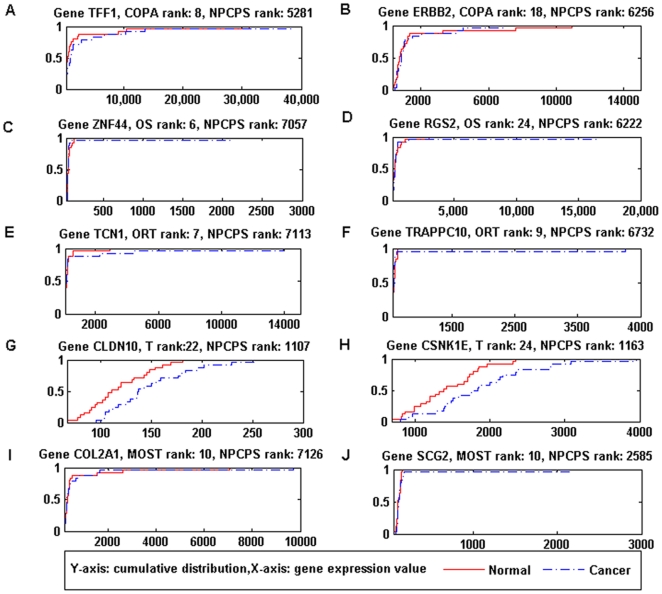
Data distributions of genes top-ranked by five parametric methods. (A) Gene TFF1, COPA rank: 8, NPCPS rank: 5281. (B) Gene ERBB2, COPA rank: 18, NPCPS rank: 6256. (C) Gene ZNF44, OS rank: 6, NPCPS rank: 7057. (D) Gene RGS2, OS rank: 24, NPCPS rank: 6222. (E) Gene TCN1, ORT rank: 7, NPCPS rank: 7113. (F) Gene TRAPPC10, ORT rank: 9, NPCPS rank: 6732. (G) Gene CLDN10, T rank: 22, NPCPS rank: 1107. (H) Gene CSNK1E, T rank: 24, NPCPS rank: 1163. (I) Gene COL2A1, MOST rank: 10, NPCPS rank: 7126. (J) Gene SCG2, MOST rank: 10, NPCPS rank: 2585. Top-ranked genes by the five parametric methods did not have significant difference between the data distributions of cancer and normal groups.

**Figure 12:**
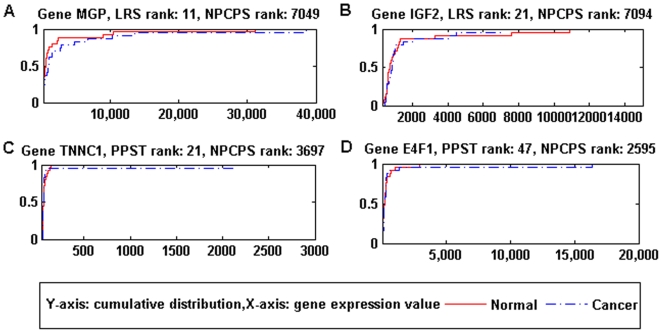
Data distributions of genes top-ranked by two non-parametric methods. (A) Gene MGP, LRS rank: 11, NPCPS rank: 7049. (B) Gene IGF2, LRS rank: 21, NPCPS rank: 7094. (C) Gene TNNC1, PPST rank: 21, NPCPS rank: 3697. (D) Gene E4F1, PPST rank: 47, NPCPS rank: 2595. Top-ranked genes by the two non-parametric methods did not have significant difference between the data distributions of cancer and normal groups.

As comparison, Fig. 11A–11J shows the data distributions of those top-ranked genes by the parametric methods, and Fig. 12A–12D by LRS and PPST. The data distributions were more similar to genes that were bottom-ranked by NPCPS in that small percent of the samples bring significant increase to data range. These few samples would greatly impact the cancer-group mean or median, which consequently result in a high test statistic of parametric methods. For example, in Fig. 11B and 12B, 96% of the two curves were close to each other while 4% data points in the normal group valued much greater, which equals to one outlier sample out of the 25 normal samples. Considering that the outliers were in the normal group, it was reasonable to assume that these outliers might be caused by microarray noise. For the rest of Fig. 11 and 12, except for T-statistic, the cancer group had one outlier. Fig. 11 and 12 indicate that the comparing methods are sensitive to significant change in mean and median, even when the change is introduced by a single sample which might be outliers. NPCPS is less prone to report a DGE as such few outliers are not sufficient to produce a large *D_n_*.

In summary, NPCPS is less sensitive to right boundaries and tends to find genes that have greater cumulative distance between the data distribution of normal and cancer groups. For such genes, the samples in normal and cancer group may have the same data range but should have very different distributions. Therefore, the detecting result of NPCPS would be different from other compared methods, which are more sensitive to outliers that influence the data range, rather than the cumulative distance between distributions. In other words, NPCPS values continuous change in data distribution over the whole data range, while the other methods look for a significant change of mean or median. This would explain the low correlation between NPCPS and other methods.

### Conclusion

A non-parametric statistical method, NPCPS, was proposed for DGE detection based on change-point theory. NPCPS uses the data distribution of normal and cancer samples as the only input to detect a change point that indicates DGE, in order to identify potential cancer genes. Distribution-based NPCPS does not require data pre-initialization and is computationally efficient compared with other median-based parametric methods. Contrast to the compared methods, NPCPS deals with both over-expression and under-expression by the same equation. Another unique feature is that the proposed NPCPS could estimate both the number and the location of cancer samples with DGE could be estimated. Simulation study and experiments showed that, the proposed NPCPS method had better reliability and accuracy; NPCPS was more effective than the compared parametric methods; similar ROC curves was given compared with LRS when sample size was larger; when the simulated DGE value was smaller, i.e. DGE was less significant, NPCPS had better sensitivity compared with the other seven methods. Simulations also indicated that, for cancer subgroup with size greater than 8, the NPCPS had FPR less than 0.01. Besides, the detection results of NPCPS had very low correlation with the compared methods, both parametric and non-parametric, which indicates that NPCPS provides meaningful detection results different from other methods. Since cancer samples could be categorized according to different stages in the cancer development, DGE detection can also be considered also a multi-class problem. Further effort could be focused on the multi-change-point in the distribution of microarray gene expression profile.
